# Underwater endoscopic submucosal resection with a ligation device for a duodenal neuroendocrine tumor

**DOI:** 10.1055/a-2742-6026

**Published:** 2025-11-28

**Authors:** Yuichiro Mikuriya, Kazuo Shiotsuki, Nobuhisa Minakata, Kohei Takizawa, Shin Maeda

**Affiliations:** 1 91321 Department of Gastroenterology, Kanagawa Cancer Center, Yokohama, Japan; 2 208511 Department of Gastroenterology, Yokohama City University Graduate School of Medicine, Yokohama, Japan

Endoscopic submucosal resection with ligation (ESMR-L) is a modified endoscopic mucosal resection technique that uses a band-ligation device, and it is effective for small duodenal neuroendocrine tumors (NETs 1 2 ). Notably, however, the tapered, tube-like shape of conventional banding devices narrows and darkens the visual field, making precise lesion capture difficult under air insufflation 3 . Water immersion can improve visualization through natural magnification and mucosal flotation 4 5 . Herein, we describe a case in which underwater ESMR-L (U-ESMR-L) provided clear visualization and enabled precise resection, with the procedure demonstrated in a video ( Video 1 ).

Underwater endoscopic submucosal resection with ligation for a small duodenal neuroendocrine tumor with enhanced visualization and precise resection.Video 1

The patient was an 83-year-old man with a 6-mm pale-yellow subepithelial lesion on the posterior wall of the duodenal bulb. Biopsy suggested a well-differentiated NET (G1). Endoscopic ultrasonography showed a submucosa-confined lesion without muscularis propria invasion, and contrast-enhanced computed tomography revealed no metastasis ( Fig. 1 ).

**Fig. 1:**
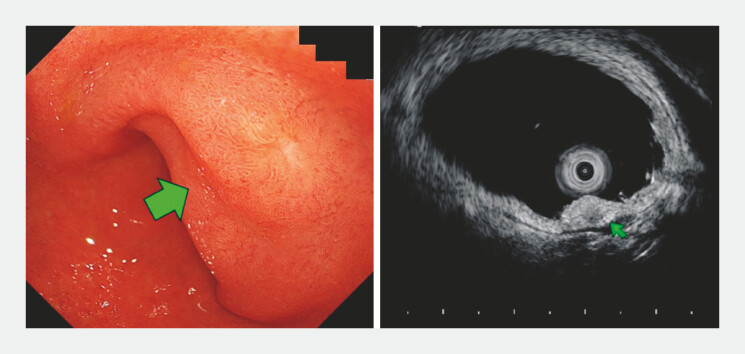
Endoscopic ultrasonography showing a submucosa-confined lesion without muscularis propria invasion. A 6-mm pale-yellow subepithelial lesion on the posterior wall of the duodenal bulb is shown.

Endoscopic resection was performed using a therapeutic endoscope (EG-840T; Fujifilm, Tokyo, Japan) with a ligation device (Pneumo-Activate EVL Device; Sumitomo Bakelite Co., Ltd, Tokyo, Japan). Under CO _2_ insufflation, the cap narrowed and darkened the endoscopic field, obscuring lesion margins. After water immersion, natural magnification and mucosal flotation enhanced clear visualization of the entire lesion ( Fig. 2 ). Gentle suction was applied to encompass the entire lesion, followed by O-ring ligation. A polypectomy snare (Polypectomy Snare H; AGS MedTech, Singapore) was then carefully positioned beneath the band, and en bloc resection was achieved via electrocautery. The defect was closed prophylactically with clips. No complications occurred, and histopathology confirmed complete resection with negative margins.

**Fig. 2:**
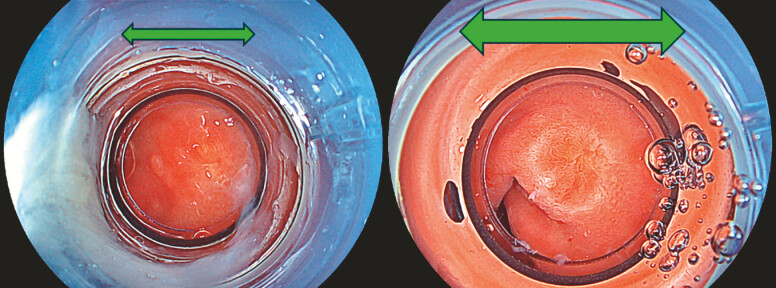
After water immersion, natural magnification and mucosal flotation enhanced clear visualization of the entire lesion.

Given that the ligation band cannot be repositioned in ESMR-L, a clear view before ligation is essential. Water immersion can improve visibility and support precise resection with ESMR-L.

Endoscopy_UCTN_Code_TTT_1AO_2AG

## Notes

Endoscopy E-Videos https://eref.thieme.de/e-videos
*E-Videos* is an open access online section of the journal *Endoscopy* , reporting on interesting cases and new techniques in gastroenterological endoscopy. All papers include a high-quality video and are published with a Creative Commons CC-BY license. Endoscopy E-Videos qualify for HINARI discounts and waivers and eligibility is automatically checked during the submission process. We grant 100% waivers to articles whose corresponding authors are based in Group A countries and 50% waivers to those who are based in Group B countries as classified by Research4Life (see: https://www.research4life.org/access/eligibility/ ). This section has its own submission website at https://mc.manuscriptcentral.com/e-videos .
